# Diagnostic and Management Strategies in Patients with Late Recurrent Angina after Coronary Artery Bypass Grafting

**DOI:** 10.1007/s11886-022-01746-w

**Published:** 2022-08-04

**Authors:** Ruben W. de Winter, Mohammed S. Rahman, Pepijn A. van Diemen, Stefan P. Schumacher, Ruurt A. Jukema, Yvemarie B. O. Somsen, Albert C. van Rossum, Niels J. Verouden, Ibrahim Danad, Ronak Delewi, Alexander Nap, Paul Knaapen

**Affiliations:** 1 Department of Cardiology Heart Center, Amsterdam UMC, Vrije Universiteit Amsterdam, Amsterdam, the Netherlands; 2 Department of Cardiology, Birmingham City Hospital, Sandwell and West Birmingham NHS Trust, Birmingham, UK; 3 Department of Cardiology Heart Center, Amsterdam UMC, Universiteit van Amsterdam, Amsterdam, The Netherlands

**Keywords:** Prior coronary artery bypass grafting, Late recurrent angina, Diagnostic algorithm, Repeat revascularization

## Abstract

**Purpose of Review:**

This review will outline the current evidence on the anatomical, functional, and physiological tools that may be applied in the evaluation of patients with late recurrent angina after coronary artery bypass grafting (CABG). Furthermore, we discuss management strategies and propose an algorithm to guide decision-making for this complex patient population.

**Recent Findings:**

Patients with prior CABG often present with late recurrent angina as a result of bypass graft failure and progression of native coronary artery disease (CAD). These patients are generally older, have a higher prevalence of comorbidities, and more complex atherosclerotic lesion morphology compared to CABG-naïve patients. In addition, guideline recommendations are based on studies in which post-CABG patients have been largely excluded.

**Summary:**

Several invasive and non-invasive diagnostic tools are currently available to assess graft patency, the hemodynamic significance of native CAD progression, left ventricular function, and myocardial viability. Such tools, in particular the latest generation coronary computed tomography angiography, are part of a systematic diagnostic work-up to guide optimal repeat revascularization strategy in patients presenting with late recurrent angina after CABG.

## Introduction

**Fig. 1:**
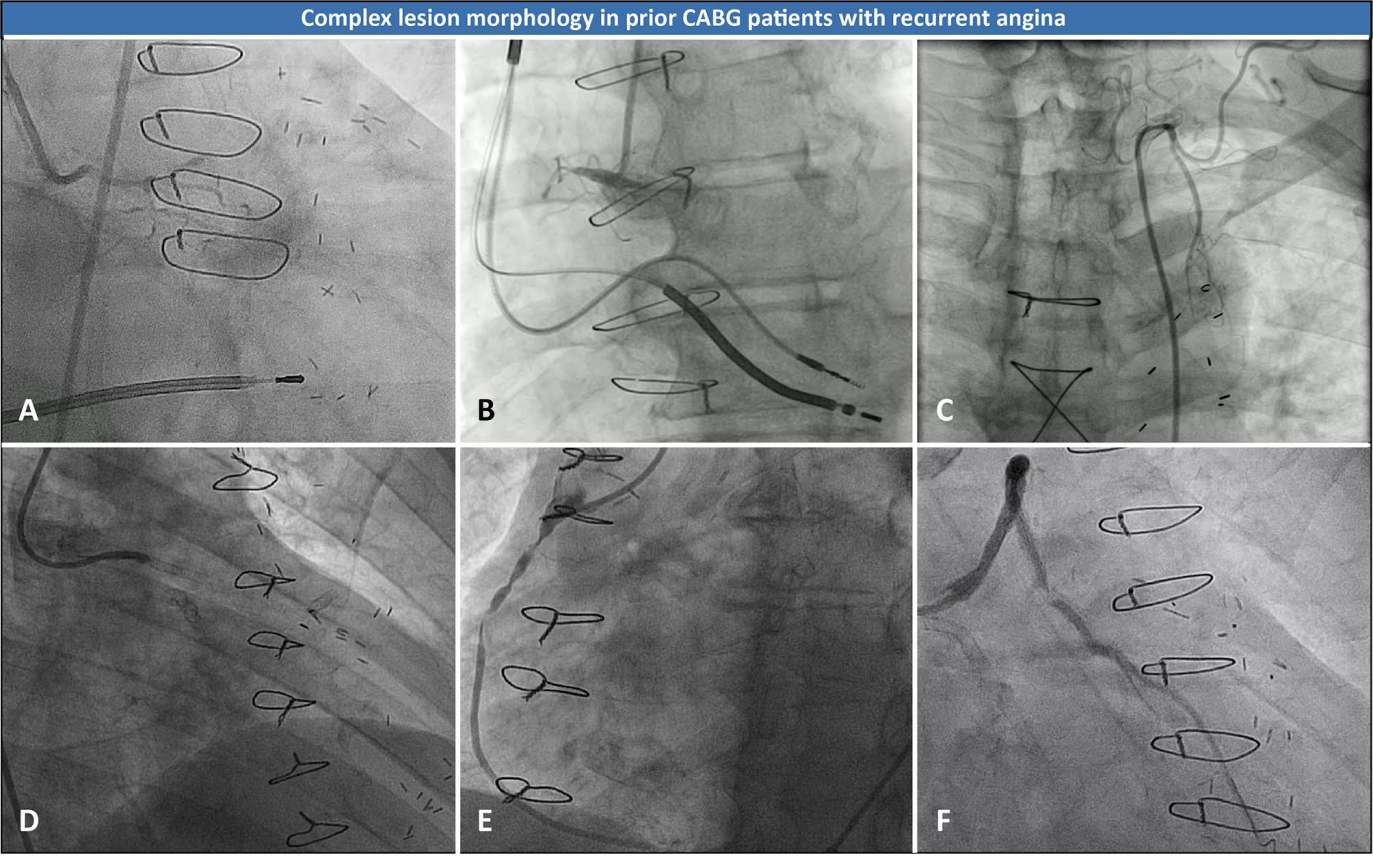
Advanced CAD and complex lesion morphology in patients presenting with late recurrent angina after CABG. Coronary angiography images showing advanced CAD and complex lesion morphology in prior CABG patients with recurrent angina symptoms. **A** Severely diseased LCA, **B** occluded RCA, **C** dysfunctional left internal mammary artery, **D** in-stent occlusion SVG on diagonal branch, **E** stenotic lesions SVG on PDA, **F** third time in-stent restenosis SVG on OM. CAD coronary artery disease, CABG coronary artery bypass grafting, LCA left coronary artery, OM obtuse marginal branch, PDA posterior descending artery, RCA right coronary artery, SVG saphenous vein graft

Coronary artery bypass grafting (CABG) effectively relieves symptoms and improves prognosis in patients with complex multivessel and/or left main coronary artery disease (CAD), particularly in patients with left ventricular dysfunction and diabetes mellitus [1•, 2•, 3]. However, despite advances in secondary prevention, a wide variety of vascular grafts available, and evolving surgical techniques, long-term efficacy of CABG is hampered by bypass graft failure and native CAD progression [4, 5]. Recurrent angina symptoms and ischemia have been reported in 18% of patients at 5 years after CABG, which increases to 40–50% at 10 years and up to 60% at 15 years postoperatively [5–7]. Indeed, many prior CABG patients undergo repeat cardiac catheterization and require subsequent revascularization therapy [8, 9••, 10]. The clinical evaluation and diagnostic work-up of patients with recurrent angina after CABG is challenging. Patients with previous CABG are generally older, have a higher prevalence of cardiac risk factors and comorbidities, more extensive CAD, and complex atherosclerotic lesion morphology (Fig. 1) [11•, 12]. Furthermore, current guideline recommendations on patient management are limited since post-CABG patients have often been underrepresented or excluded in large diagnostic and revascularization trials [1•, 2•, 10, 13, 14•, 15•]. Late recurrent angina in patients with prior CABG is common and often requires individualized decision-making by the Heart Team. Information on graft anatomy, ischemia, viability, presence of scar tissue, and ventricular function collected prior to invasive coronary angiography (ICA) can guide management strategies [15•]. This review will outline the available evidence on non-invasive and invasive diagnostic tools to evaluate bypass graft failure and CAD progression while considering clinical characteristics in these complex patients. In addition, we discuss potential treatment strategies and propose an algorithm to guide decision-making at the outpatient clinic.

## Clinical Evaluation of Patients with Recurrent Angina After CABG

### Time Interval Between CABG and Recurrent Symptoms

The timing of recurrent angina after CABG is important in understanding its underlying etiology and can be divided into early (< 1 month), intermediate (1 month–1 year), and late (≥ 1 year) recurrence [16]. Early graft failure predominantly results from acute thrombotic complications, technical issues regarding graft harvesting, or imperfect sutures at the site of the newly constructed anastomoses [17, 18••, 19]. Contributing factors to early graft attrition include poor distal runoff, conduit size mismatch, pre-existing graft disease, and low-grade stenosis proximally in the grafted native coronary artery causing competitive flow in the bypass conduit [17, 18••]. After the first month following bypass surgery, recurrence of angina may be related to obstructive lesions at the graft anastomosis or incomplete revascularization [16]. Additionally, progressive neointimal hyperplasia and endothelial damage may occur in saphenous vein grafts (SVG) secondary to surgical trauma and arterial systemic pressure related shear stress [20]. This initiates a cascade of cytokine release, proliferation of smooth muscle cells and fibrotic changes, resulting in luminal loss and graft failure [18••, 21]. Finally, late recurrent angina following CABG is often related to degenerative atherosclerotic disease and intimal hyperplasia resulting in bypass graft failure, which is mainly observed in venous conduits [16, 22]. Furthermore, progression of native CAD may result in new lesions causing ischemia in non-grafted coronary arteries or grafted vessels distal to the anastomosis.

### Vascular Conduits and Long-Term Patency

When evaluating the likelihood of graft failure in patients presenting with late recurrent angina after CABG, it is essential to consider the number, origin, and sequence of vascular conduits. Several vascular conduits are currently available: the left and right internal mammary artery (LIMA/RIMA), radial artery (RA), gastroepiploic artery, and venous bypass grafts. In contemporary practice, the LIMA is advocated to revascularize the left anterior descending (LAD) coronary artery, whereas SVGs are mostly utilized to bypass non-LAD territories [23, 24]. IMA grafts demonstrated excellent patency rates between 85 and 91% 10 years after CABG, with comparable patency rates for the LIMA and RIMA used to bypass the left coronary system [18••]. Furthermore, no differences in graft patency were observed in patients with bilateral arterial grafting with both IMA grafts using the RIMA in-situ or in an Y-grafting configuration [25]. Similar findings have been reported regarding the use of the RA as an Y-graft [26]. In addition, the RADIAL (Radial-Artery or Saphenous-Vein Grafts in Coronary-Artery Bypass Surgery) trial reported a 5 year patency of 92% for the RA graft, whereas long-term results of the RAPCO (Radial Artery Patency and Clinical Outcomes) trial showed a 89% patency rate at 10 years [27, 28]. The use of the gastroepiploic artery graft is decreasing due to its technical complexity and because patency at 3 years has been demonstrated to be only 60% [29••]. In spite of this large body of evidence demonstrating lasting arterial graft patency, SVGs remain the most common conduits in coronary bypass surgery, used in approximately 80–90% of patients [23, 30]. Saphenous vein graft failure has been reported between 10 and 20% within 1 year after CABG, with an additional yearly failure rate of 1–2% between 1 and 6 years [19, 29••]. Hereafter, SVG failure rates accelerate, resulting in approximately 50% graft patency at 10 years after CABG [31]. There is no consensus in the literature on the superiority in terms of patency rates and adverse outcome for the use of SVGs with a single distal anastomosis compared to using a so called ‘jump,’ ‘snake,’ or ‘sequential’ vein graft with 1 end-to-side anastomosis and 1 or more side-to-side anastomoses [32]. Although improvements in surgical techniques are expected to increase SVG patency (particularly no-touch SVG harvesting yields promising results), SVG failure is anticipated to remain a dominant cause of late recurrent symptoms after CABG [24, 29••].

### Clinical Presentation

Chronic coronary syndrome patients with late recurrent angina after CABG may recognize their symptoms from a previous symptomatic episode before the initial bypass surgery. Nevertheless, in a substantial proportion of post-CABG patients, recurrent angina symptoms are atypical and assessment of the causal relationship with underlying myocardial ischemia can be challenging [33, 34]. Herlitz et al. found that half of the post-CABG patients experienced recurrence of chest pain symptoms, whereas more than two-third reported dyspnea, suggested to be partly related to chronic heart failure, patient age, and comorbidities [35]. Naturally, prior CABG patients presenting with angina equivalents, rhythm abnormalities or deterioration of left ventricular function may also require additional diagnostic work-up and repeat revascularization therapy [36]. Of note, whereas IMA graft failure rarely occurs without causing ischemic symptoms, venous bypass graft failure may be observed during per protocol performed coronary angiography in asymptomatic patients [37–39]. This phenomenon may be explained by a small myocardial territory supplied by the venous graft, the grafting of vessels with non-obstructive lesions at index surgery, or collateral donor arteries supplying the myocardial territory of the failing bypass graft [18••]. Whether symptomatic or asymptomatic, bypass graft failure has been associated with adverse patient outcome [36, 39–42].

## Diagnostic Evaluation and Non-Invasive Imaging in Patients with Prior CABG

Recurrent symptoms in post-CABG patients may be related to a broad spectrum of non-coronary and non-cardiac disease [16]. Therefore, a thorough clinical and systematic diagnostic evaluation is vital to establish symptom etiology. Firstly, evaluation of the clinical presentation with a thorough patient history should be taken, including consideration of prior illnesses, cardiovascular risk factors, comorbidities, and the clinical reports of index bypass surgery in combination with previous coronary angiography images if available, to assess the origin of vascular conduits and the number of distal anastomoses. The next step in the work-up is basic assessment with laboratory tests, a resting electrocardiogram, and echocardiography to assess left ventricular ejection fraction and possible valve dysfunction [14•]. After this primary survey, additional non-invasive imaging can help elucidate whether or not recurrent symptoms are related to underlying obstructive coronary artery or graft disease. ICA is considered the reference of care for evaluation of significant CAD in patients with prior CABG and a high clinical probability of ischemic heart disease as the cause of recurrent symptoms [14•]. Therefore, the diagnostic accuracy of non-invasive imaging tools has been mostly related to ICA as the gold standard. However, ICA exposes patients to the inherent risk of complications, radiation and contrast burden, considerable costs, and discomfort [43, 44]. Furthermore, it has been suggested that the risks of ICA are higher in patients with a history of CABG compared to CABG-naïve patients, which is in part due to higher incidence of comorbidities, increased fluoroscopy times, and the risk of embolization and dissection during catheter manipulation to engage bypass grafts [45, 46]. Currently, technical advances have led to an increased interest in non-invasive imaging as an alternative for ICA to reduce the number of patients referred to the cardiac catheterization laboratory that do not have lesions that require further revascularization therapy (reported to occur in 40% of post-CABG patients) and to subsequently guide invasive management [47, 48]. Whereas the current European Society of Cardiology (ESC) chronic coronary syndrome guideline recommendations do not distinguish between patients with and without prior CABG, the recently published AHA/ACC/ASE/CHEST/SAEM/SCCT/SCMR Guideline for the Evaluation and Diagnosis of Chest Pain recommends a more prominent role for coronary computed tomography angiography (CCTA) and stress testing in post-CABG patients (level of evidence 2A) [14•, 15•]. Importantly, stress testing in patients with prior CABG has limitations. Coronary microvascular dysfunction, which has been related to cardiovascular risk factors and advanced atherosclerosis (frequently observed in prior CABG patients), can cause myocardial ischemia during stress testing in the absence of obstructive epicardial coronary disease. One should bear in mind that myocardial perfusion imaging (MPI) values in patients with prior cardiac history may be globally reduced solely due to diffuse CAD and coronary microvascular disease, which negatively impacts the specificity to detect significant graft failure and/or progression of native epicardial lesions [16, 49–52]. Furthermore, chronic coronary syndrome guidelines do not list recommendations on the preferred stress test for patients with recurrent angina ≥ 1 year after initial revascularization, thus application is based on patient characteristics, clinical likelihood, local expertise, availability, and individual clinician preference. Below, we provide an outline of the current evidence, advantages, and disadvantages of the available non-invasive and invasive tools for the anatomical, functional, and physiological assessment of patients with recurrent angina after CABG (Table 1).

**Table 1:** Diagnostic modalities for the evaluation of late recurrent angina after CABG

Diagnostic modality	Characteristics
**Stress electrocardiography **[14•, 118]	
*Advantages*	Widely available
	Low-cost
	Provides information on exercise tolerance and risk stratification
*Disadvantages*	Lacks sufficient diagnostic accuracy
	Stress imaging favored to more accurately establish the site and extent of ischemia
**Stress echocardiography **[16, 119]	
*Advantages*	Low-cost
	Easily accessible tool for assessment of myocardial perfusion at the bedside without radiation burden
*Disadvantages*	Noise and artefacts are common
	Lack of reproducibility
	Variable image quality and time-consuming manual analysis
**CCTA **[55••, 59–66, 72, 74]	
*Advantages*	First and sometimes only diagnostic test
	Widely available
	Procedural (CTO PCI) planning
	A large number of studies showed that the patency of coronary bypass grafts can be accurately assessed by CCTA due to the large diameter, reduced susceptibility to motion along the cardiac cycle and minimal calcification
*Disadvantages*	Reduced diagnostic accuracy native CAD and distal anastomoses in the presence of severe atherosclerotic disease (artefacts due to heavy calcification/prior PCI with stenting) and relatively small diameter of the distal vessels
	(Current) Lack of functional assessment
**CMR **[75, 76]	
*Advantages*	Anatomical imaging combined with physiological assessment of blood flow and myocardial perfusion to evaluate vessel morphology + functional status simultaneously without radiation burden
	Scar/viability assessment
*Disadvantages*	Safety and imaging quality may be impeded by metallic implants such as ostial graft markers, sternal wires, CABG clips, implantable electronic devices, prior stents, calcification, and heart valve prostheses
**SPECT **[84•]	
*Advantages*	Widely available
	Assessment of the extent of myocardial ischemia as a percentage of the left ventricle Global/regional left ventricular function + scar assessment
*Disadvantages*	Moderate image quality owing to attenuation and low spatial resolution
	Conventional SPECT relies on relative uptake images without absolute quantification of MBF in mL min^−1^ g^−1^
	Limited evaluation of patients with extensive CAD, three vessel disease, left main disease and microvascular dysfunction
**PET **[84•]	
*Advantages*	Excellent resolution properties and technically best suited for myocardial blood flow quantification
*Disadvantages*	High costs
	Limited availability
**Invasive coronary angiography **[14•, 43–46]	
*Advantages*	Reference standard for evaluation of obstructive CAD and graft disease
	Possibility to treat in the same session
	Prior testing for procedural guidance which may result in lower contrast volumes, reduced ionic radiation doses and faster procedural times
*Disadvantages*	Inherent (small) risk of complications
	Considerable costs
	Relatively high radiation burden + large contrast volume (particularly in post-CABG patients)
	Uncertain value of invasive physiological assessment^a^

*CABG* coronary artery bypass grafting, *CAD* coronary artery disease, *CCTA* coronary computed tomography angiography, *CMR* cardiac magnetic resonance imaging, *CTO* chronic total coronary occlusion, *ECG* electrocardiogram, *PCI* percutaneous coronary intervention, *PET* positron emission tomography, *SPECT* single-photon emission computed tomography

^a^The evidence for the use of invasive functional testing in patients with prior CABG is scarce

### Coronary Computed Tomography Angiography

**Fig. 2:**
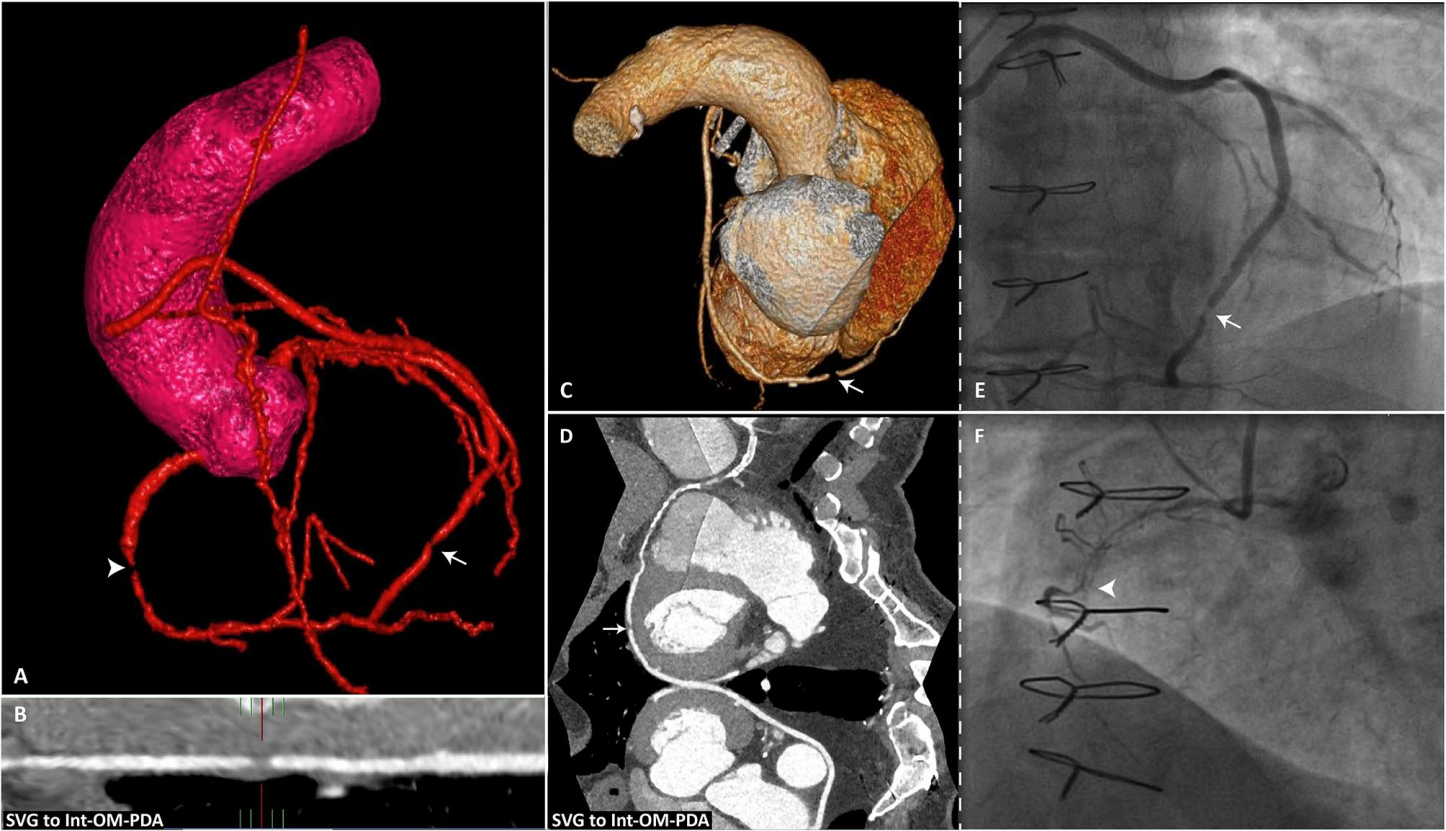
CCTA to evaluate bypass graft failure. CCTA and subsequent invasive coronary angiography images in a post-CABG patient with recurrent angina symptoms. **A**–**D** CCTA reconstructed images showing a significant lesion in a saphenous vein jump graft with distal anastomoses on the intermediate branch, an OM branch and the PDA. The lesion is located between the anastomosis on the OM branch and the PDA (white arrows). In addition, a CTO was found in the RCA, depicted in the 3-dimensional reconstruction image in (**A**), indicated with the white arrowhead. **E** Subsequent angiography images confirmed the lesion in the venous jump graft (white arrow) (**F**) and in stent occlusion of the RCA (white arrowhead). CCTA coronary computed tomography angiography, CTO chronic total coronary occlusion; other abbreviations as in Fig. 1

In patients with prior CABG, the potential applicability of CCTA to serve as a viable alternative for ICA and rule out bypass graft failure is becoming increasingly evident. The American multi-societal appropriate use criteria for cardiac CT advocate the use of CCTA to evaluate patients with stable recurrent symptoms due to suspected ischemia, in particular to examine graft patency [53]. A meta-analysis by Barbero et al. examined 959 patients with 2482 bypass grafts and reported excellent sensitivity (98%; 95% CI 0.97–0.99) and specificity (98%; 95% CI 0.96–0.98) for detection of obstructive graft lesions (> 50% diameter stenosis on ICA). These findings were regardless of age and consistent in both arterial and venous conduits resulting in an area under the curve of 0.99 [54]. Compared to native coronary arteries, coronary artery bypass grafts are larger in diameter, less susceptible to motion along the cardiac cycle, and minimally calcified, which contributes to the high accuracy of CCTA imaging to detect significant graft disease [55••]. The introduction of third-generation dual-source CT scanners with higher temporal and spatial resolution has resulted in excellent image quality, detailed graft visualization, and lower radiation dose [47, 56]. Recently, Mushtaq et al. used a new generation, 256-slice CT scanner, and described an overall diagnostic accuracy of 100% to assess graft patency, which was similar for all graft segments irrespective of cardiac rhythm and heart rate [57•]. This may be especially relevant since atrial fibrillation is present in 20–40% of post-CABG patients [58]. Figure 2 illustrates the potential of CCTA to evaluate graft failure in a patient with recurrent angina after CABG. Detection of significant stenosis at the site of graft anastomosis or in native coronary arteries may be more challenging in post-CABG patients due to advanced and diffuse native coronary atherosclerotic disease, severe calcification, high incidence of prior percutaneous coronary intervention (PCI) with stenting, and the relatively small diameter of the vessels distal to the anastomoses [55••, 59, 60]. Prior studies in recipient and non-grafted coronary arteries in patients with previous CABG demonstrated sensitivity in the range of 83–100%, whereas specificity was reported between 76 and 97% to detect significant luminal stenosis > 50% on ICA [60–63]. CCTA has the tendency to overestimate lesion severity due to calcification induced blooming and partial volume artefacts, which hamper specificity [64, 65]. Even when CCTA results are inconclusive and are unable to exclude significant graft disease and/or progression of native CAD as the potential cause of recurrent angina, the derived anatomical information still provides key information in patients who undergo subsequent ICA. CCTA can enhance the understanding of graft anatomy and guide procedural planning for invasive management in post-CABG patients, particularly when there is an uncertain number of grafts or unclear aorto-ostial location. In addition, assessment of graft patency by CCTA is associated with reduced contrast volumes, lower ionic radiation burden, and faster procedural times during subsequent ICA, compared to patients without prior CCTA imaging [66]. However, it remains uncertain if the reduction of fluoroscopy time and contrast dose during ICA is sufficient to level out additional exposure during CCTA. Currently, the GREECE (Computed Tomography Guided Invasive Coronary Angiography in Patients with a Previous Coronary Artery Bypass Graft Surgery) randomized trial is recruiting patients aiming to compare radiation and contrast burden in patients with and without prior CCTA undergoing diagnostic ICA [67]. Furthermore, the randomized BYPASS-CTCA (the Value of Computed Tomography Cardiac Angiography in Improving Patient-related Outcomes in Patients with Previous Bypass Operation Undergoing Invasive Coronary Angiography) study aims to include 688 participants and hypothesizes that CCTA prior to ICA may reduce contrast-induced kidney injury and procedural times [45]. Apart from the potential diagnostic implications of CCTA in patients with prior CABG, several studies illustrated its value for long-term risk stratification by classification of protected and unprotected coronary territories according to graft patency and obstructive native vessel CAD [68, 69]. Furthermore, cardiac CT may aid procedural planning for re-sternotomy to assess mediastinal adhesions and graft anatomy to limit the risk of complications during sternal reentry [55••]. Some reports have been published on myocardial ischemia due to a stenosis of the subclavian artery in the presence of an IMA graft, which can also be appreciated with CCTA [70, 71]. Finally, results from the CT-RECTOR (Computed Tomography Registry of Chronic Total Occlusion Revascularization) registry suggested that CCTA imaging prior to revascularization of a chronic total coronary occlusion (CTO), which are observed in > 50% of post-CABG patients referred to the catheterization laboratory, may predict effective guide-wire crossing and optimize procedural times [72, 73]. Studies on functional CT modalities such as CT perfusion and CT-based fractional flow reserve (CT-FFR) have not included prior CABG patients and its additive value remains to be established [74]. In summary and in concordance with the recently published Society of Cardiovascular Computed Tomography 2021 expert consensus document, CCTA is a useful first tool in the evaluation of patients with prior CABG and ischemic symptoms, particularly recommended to evaluate graft patency [55••].

### Cardiovascular Magnetic Resonance Imaging

Cardiovascular magnetic resonance imaging (CMR) is a non-invasive tool to acquire static or dynamic images of the heart and large vessels without the use of ionizing radiation. CMR safety and imaging quality in prior CABG patients may be impeded by metallic implants such as ostial graft markers, sternal wires, CABG clips, prior stents, implantable electronic devices, and heart valve prostheses [75]. However, CMR holds the advantage that anatomic/angiographic imaging can be combined with physiological assessment to evaluate graft patency and functional status simultaneously [76]. Several studies explored the applicability of CMR for the anatomical assessment of bypass graft patency. Langerak et al. used high-resolution navigator-gated 3-dimensional MR angiography to assess vein graft patency in 38 patients (including 56 bypass conduits) presenting with recurrent chest pain symptoms and reported a sensitivity of 83% (95% CI 0.36–1.00) and specificity of 100% (95% CI 0.92–1.00) for the assessment of graft occlusion. Graft stenosis of ≥ 50% could be diagnosed with a sensitivity of 82% (95% CI 0.57–0.96) and specificity of 88% (95% CI 0.72–0.97), yet only after excluding patients with stents and scans with limited image quality (11%) [77]. Additionally, in a proof-of-concept study, Langerak and colleagues evaluated the diagnostic accuracy of CMR combining MR angiography and flow velocity mapping to detect obstructive lesions in venous conduits (*N* = 125) and their recipient coronary arteries [48]. They found a sensitivity of 94% and specificity of 63% for the detection of single vein graft stenosis ≥ 50%, after excluding 20% of unsuccessful stress scans related to adenosine-induced side-effects. A meta-analysis by Dikkers et al. compared the diagnostic accuracy of CMR and CCTA to detect graft occlusion and stenosis of both arterial and venous conduits. The authors reported an overall sensitivity of 81% (95% CI 0.76–0.86) and specificity of 91% (95% CI 0.89–0.93) for the evaluation of graft occlusion with CMR in a pooled analysis of 19 studies. This meta-analysis included only 2 studies that explored the value of CMR to detect bypass graft stenosis and noted a 86% sensitivity and 94% specificity for IMA grafts stenosis ≥ 70%, whereas a sensitivity of 62% and specificity of 82% were documented for ≥ 50% stenosis in a combined dataset with 12 arterial and 45 venous conduits [78]. Notably, the majority of studies exploring the value of perfusion CMR for detection and characterization of ischemic myocardium excluded patients with prior CABG, which may be related to altered coronary hemodynamics in these patients [79]; indeed, myocardial blood flow (MBF) patterns, contrast kinetics, and perfusion properties may vary between different bypass grafts and between grafts compared to native coronary arteries, yet the impact of these differences on the diagnostic accuracy of first-pass perfusion CMR remains controversial [80–82]. One study by Bernhardt et al. scheduled 110 patients referred for ICA to undergo prior stress perfusion CMR integrated with late gadolinium enhancement and reported 79% sensitivity and 77% specificity for detecting coronary artery or bypass graft stenosis using ≥ 70% stenosis on ICA as a reference, which was significantly lower compared to CABG-naïve patients [83]. Thus, CMR as a non-invasive alternative for ICA with the sole aim to assess graft patency seems inferior to CCTA. Still, CMR may provide additive information on inducible myocardial ischemia and scar tissue, contributing to the versatility of CMR in the non-invasive assessment of patients with prior CABG.

### Single-Photon Emission Computed Tomography

**Fig. 3:**
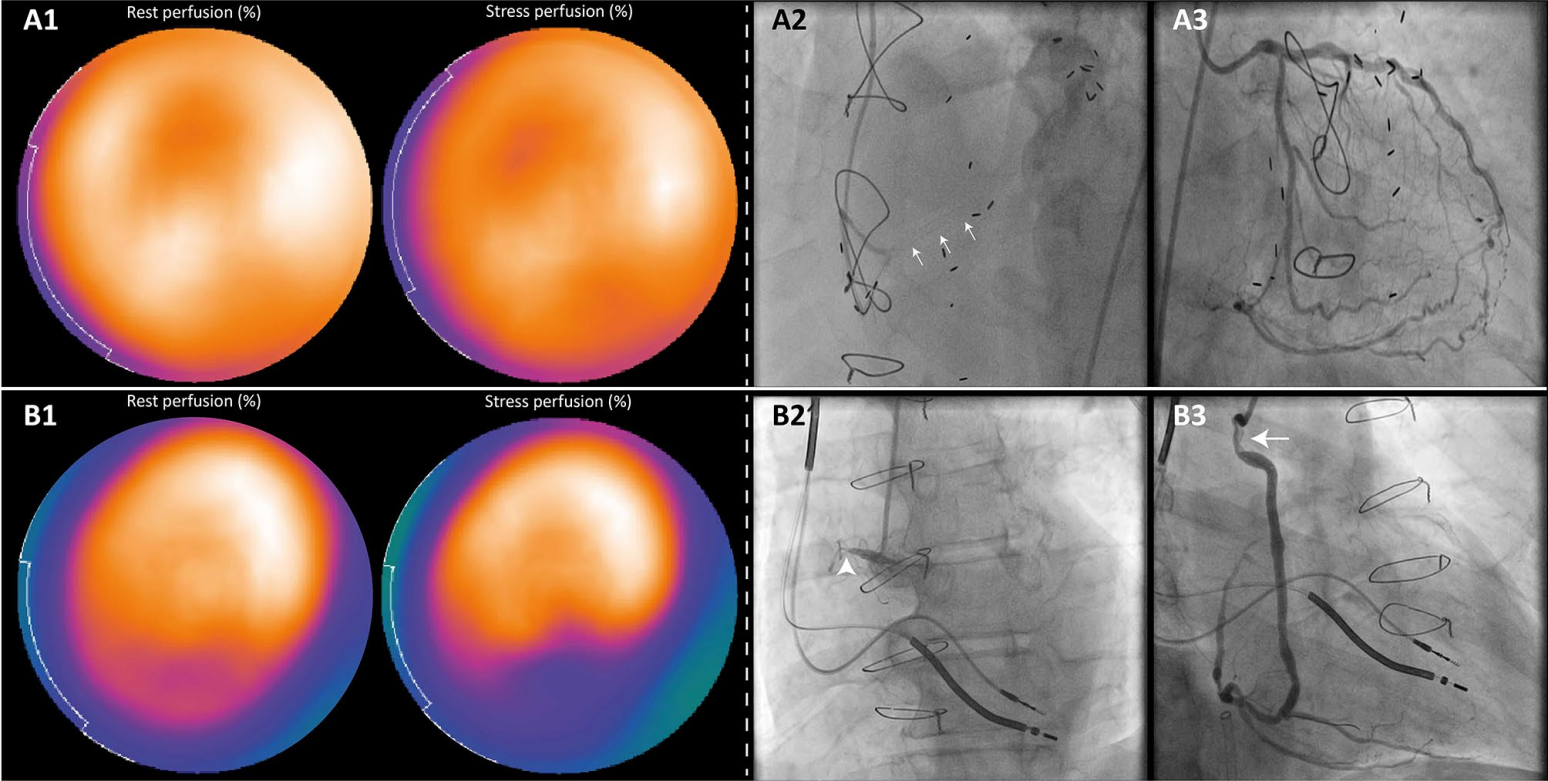
SPECT perfusion imaging in patients with previous CABG. Rest and stress (intravenous adenosine 140 mg/kg/min) SPECT perfusion results acquired during a 2-day protocol using a weight-adjusted dose (370 to 550 MBq) of.^99m^Tc tetrofosmin as a radiopharmaceutical, in conjunction with ICA images are illustrated. **A1** SPECT-MPI in a patient presenting with dyspnea on exertion (RDS 2) and index bypass surgery 14 years ago showed mildly reduced tracer-uptake in the distal and mid anterior segments which persisted during hyperemia (differential diagnosis: a prior MI or an attenuation artefact). **A2** Angiography showed a small caliber single SVG on the PDA with chronic in-stent occlusion of the distal native PDA indicated with the white arrows. **A3** Extensive collateral circulation from the LCA supplied the occluded PDA (Rentrop grade 3, CC score 2). Despite the presence of well-developed collaterals, myocardial ischemia in the CTO territory is observed in over 90% of patients [115]. However, SPECT did not reveal ischemia in the inferior wall and may have been false negative. **B1** Perfusion scintigraphy in a patient presenting with stable angina symptoms (CCS 4) 42 years after CABG revealed reduced tracer uptake in the inferior/inferolateral mid and basal segments which expanded during stress. **B2**, **B3** ICA showed a CTO of the RCA (white arrowhead) and an obstructive stenosis in the proximal part of the SVG on the PDA (white arrow). SPECT-MPI results were therefore graded true positive. CC collateral connection, CCS Canadian Cardiovascular Society Angina Score, ICA invasive coronary angiography, MBq megabecquerel, MI myocardial infarction, MPI myocardial perfusion imaging, RDS Rose Dyspnea Scale, SPECT single-photon emission computed tomography; other abbreviations as in Figs. 1 and 2

Single-photon emission computed tomography (SPECT) is the most commonly used MPI tool. SPECT has proven to accurately assess the extent of myocardial ischemia as a percentage of the total left ventricle and is used to evaluate global and regional left ventricular function [84•]. In addition, SPECT may provide incremental prognostic information in patients with stable CAD. Indeed, the concept that patients with a large area of myocardial ischemia (> 10% of the left ventricle) may benefit from revascularization compared to optimal medical therapy alone is based on large SPECT MPI studies and is still used to guide clinical decision-making [1•]. Conventional SPECT MPI is widely available but may be of limited value in the evaluation of patients with extensive CAD/three-vessel disease, left main disease, and microvascular dysfunction since it relies on relative differences in regional perfusion and perfusion defects may not be evident in patients with balanced ischemia [85, 86]. However, recent technological advances may allow for SPECT quantification of MBF and myocardial flow reserve, increasing its value for evaluation of recurrent ischemia in patients with complex CAD despite the limited spatial resolution [84•]. Several studies explored the application of SPECT in the diagnostic work-up of patients with recurrent symptoms after CABG. Lakkis et al. evaluated 119 grafts with Thallium-21 SPECT approximately 1 year after CABG in symptomatic patients to detect obstructive graft disease and reported sensitivity of 84% and specificity of 80% in patients with typical angina [87]. In a study by Khoury et al. including 109 patients with recurrent symptoms (283 bypass grafts), SPECT MPI was performed 6.7 ± 4.8 years after CABG and a sensitivity of 96% was observed for detecting significant graft disease, whereas the specificity was only 61%. The authors suggested that the high rate of false positive findings in their cohort was largely attributed to persistent perfusion defects in patients with prior MI or by progression of native CAD in non-grafted myocardial territories [88]. Elhendy et al. evaluated 71 patients with dobutamine SPECT with a median time of 3.7 years between surgery and scan acquisition and described a sensitivity, specificity, and diagnostic accuracy of 81%, 79% and 80%, respectively, to detect significant lesions of ≥ 50% diameter stenosis of both bypass grafts and non-grafted coronary arteries. The sensitivity was 64% to detect isolated graft lesions, whereas specificity was 85% [89]. As such, stress testing with SPECT MPI in patients with late recurrent symptoms and a history of CABG may distinguish angina symptoms related to myocardial ischemia or an alternative non-cardiac diagnosis, predict graft disease with moderate accuracy, and estimate individual patient risk to guide revascularization strategies. Several limitations should be taken into account when interpreting SPECT findings since a negative result may be related to the inability to detect relative perfusion defects in patients with globally reduced perfusion values due to extensive atherosclerotic disease, whereas perfusion abnormalities in the absence of flow limiting stenosis in the graft and/or native coronary arteries may be related to coronary microvascular disease or previous MI and scar (Fig. 3).

### Positron Emission Tomography

Positron emission tomography (PET) is the non-invasive reference standard for absolute flow quantification, which enables the detection of global ischemia [84•]. Multiple PET tracers such as ^82^Rb, ^13^NH_3_, and [^15^O]H_2_O are available for perfusion imaging and the application of PET MPI is expected to expand in the coming years [84•]. Limited evidence is currently available on the value of PET MPI to assess graft patency or progression of CAD in patients with a history of CABG and late recurrent symptoms. Importantly, the use of PET has been described to independently predict three-vessel disease, adding incremental value over relative perfusion assessment alone [90]. This can be relevant in patients with prior CABG since it has been suggested that coronary vasodilator reserve, assessed by ^13^NH_3_ PET MPI, is attenuated even in myocardium subtended by patent bypass grafts [91]. One study published in 1992 compared Thallium SPECT with ^82^Rb PET MPI and described superior sensitivity for PET (93% vs. 76%) for the detection of graft disease or progression of native CAD 6.5 years after CABG [92]. However, contemporary trials comparing PET with SPECT performed with modern tracers are not available. Future larger studies are warranted to establish the additional value of quantification of MBF and flow reserve by PET in patients with prior CABG.

### Hybrid Imaging

**Fig. 4:**
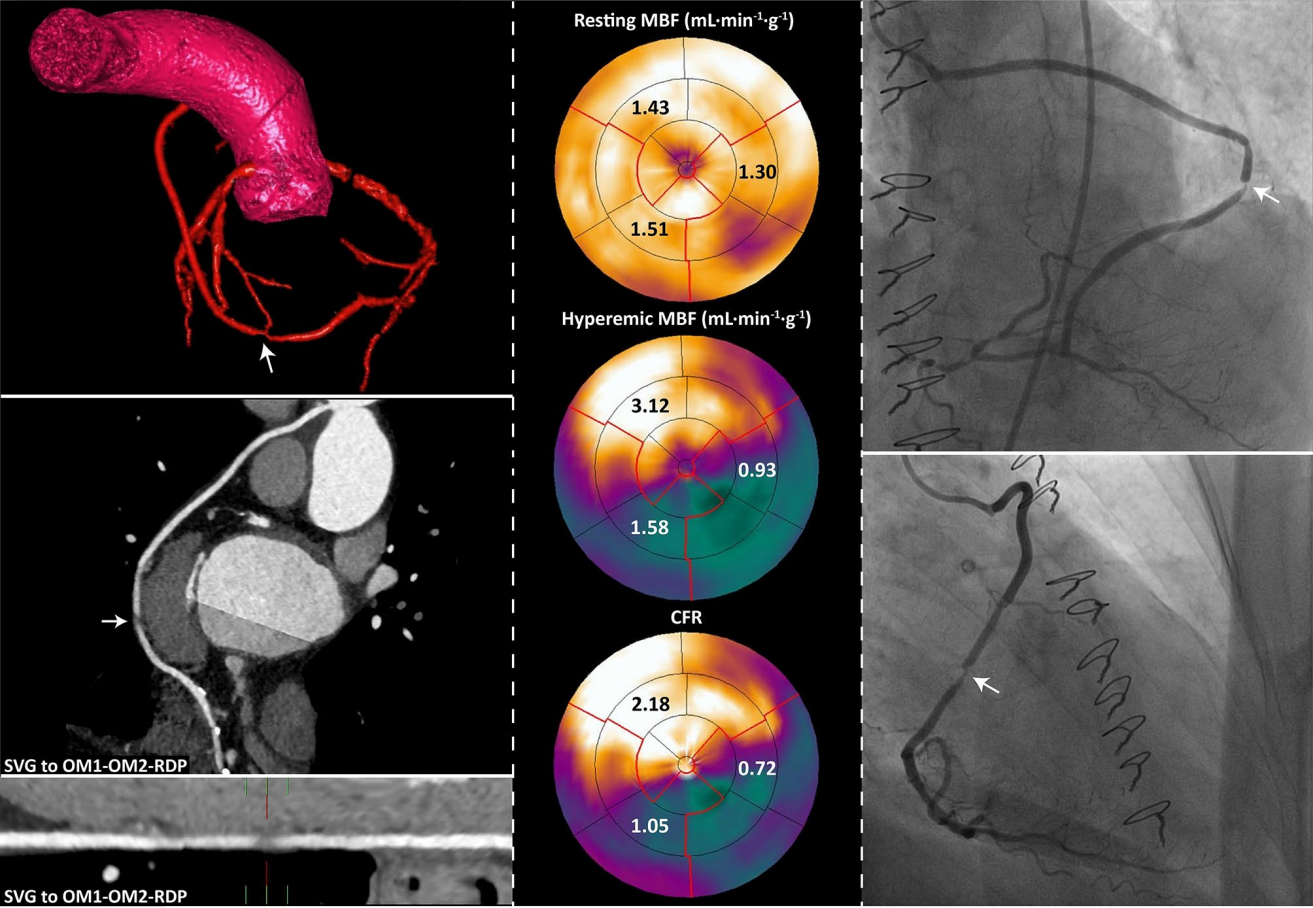
Hybrid imaging to predict hemodynamically significant bypass graft lesions. The left panels show CCTA images of a post-CABG patient with recurrent angina and a saphenous vein jump graft on the first and second OM branches and the PDA. A lesion in the vein graft was observed located at the second anastomosis with the OM2 branch (white arrows). [^15^O]H_2_O PET perfusion imaging showed myocardial ischemia in the LCx (hyperemic MBF 0.93 mL min^−1^ g^−1^ and CFR 0.72) and RCA coronary vascular territories (hyperemic MBF 1.58 mL min^−1^ g.^−1^ and CFR 1.05). The combined anatomical and functional information can assist the Heart Team to decide to refer a patient to the catheterization laboratory or rather select a conservative strategy. This patient was referred for subsequent ICA, which confirmed a lesion in the SVG as the possible cause of ischemia. CFR coronary flow reserve, LCx left circumflex coronary artery, MBF myocardial blood flow, PET positron emission tomography; other abbreviations as in Figs. 1, 2, and 3

In a study by Maaniitty et al., [^15^O]H_2_O PET perfusion imaging and CCTA were used to assess the additive value of combined functional and anatomical information in patients with recurrent symptoms after CABG [93]. The authors studied 36 patients and illustrated how the use of hybrid imaging may allow for the evaluation of perfusion abnormalities in protected and unprotected coronary territories as established with CCTA. However, the sample size was small, invasive coronary angiography to provide a reference standard was not performed, and patient outcome was not reported. In another small observational study performed by Kawai et al., the use of combined CCTA/SPECT imaging was shown to improve the prediction of adverse cardiac events in patients with prior CABG [94]. Although large, prospective trials are lacking, the non-invasive consideration of ischemia in conjunction with information on graft patency may assist the Heart Team to decide to refer a patient to the catheterization laboratory or rather select a guideline-directed optimal medical strategy (Fig. 4).

## Invasive Management in Patients with Prior CABG

### Invasive Coronary Angiography and Coronary Physiology

**Fig. 5:**
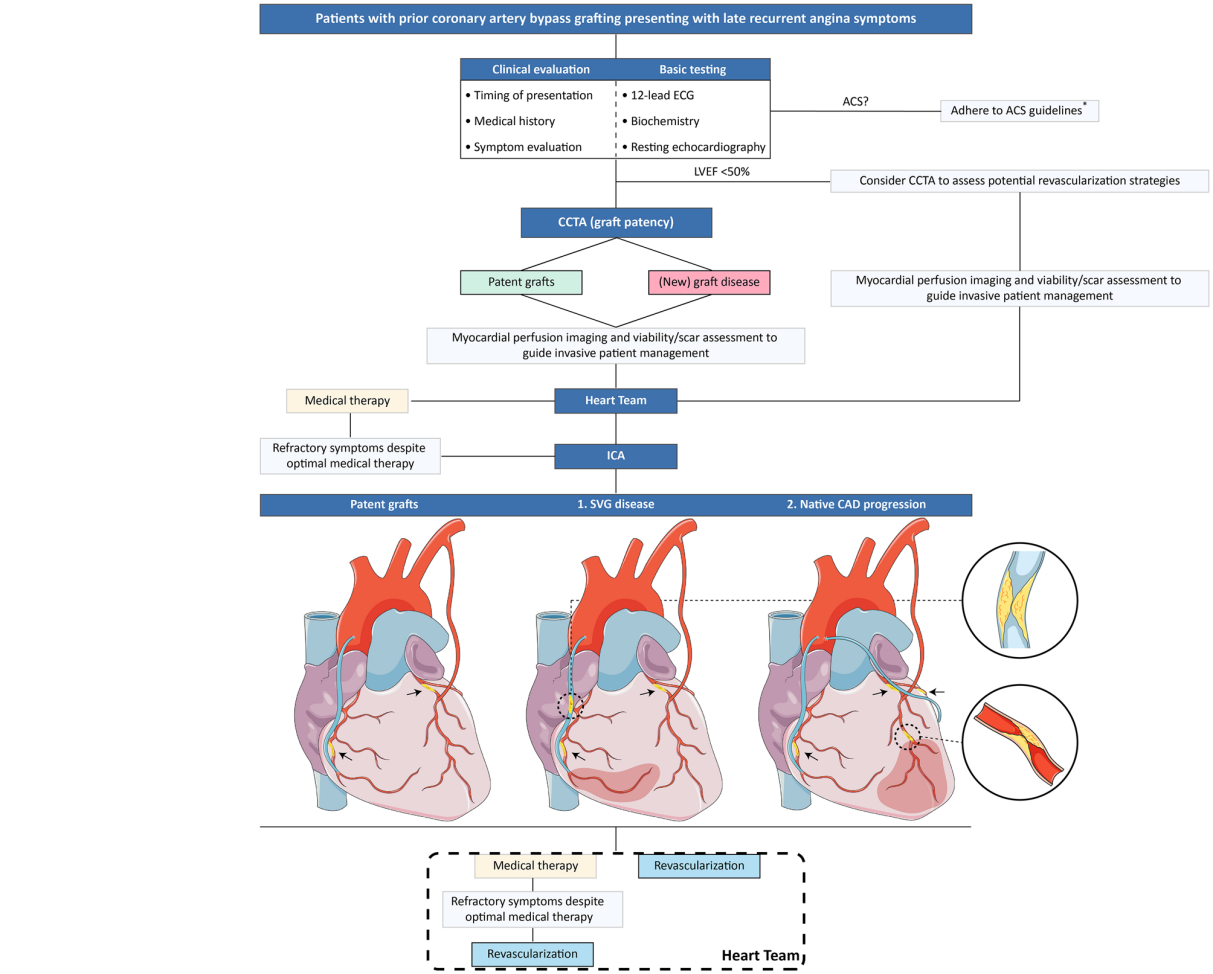
Management algorithm for patients with late recurrent angina after CABG_._ We propose an algorithm for the outpatient clinic to guide decision-making for patients presenting with late recurrent angina after CABG. A central role is depicted for CCTA, particularly to assess graft patency. Ideally, the Heart Team is provided with information on the timing of recurrent symptoms after initial CABG, cardiac history (including details on left ventricular function and valve abnormalities), CCTA images to establish protected and unprotected coronary territories, and stress testing results to evaluate myocardial ischemia and scar/viability if indicated, to guide revascularization decision-making. The use of the algorithm at the outpatient clinic can be illustrated by 2 example cases. Case 1. A patient with a history of CABG (2002) presented at the outpatient clinic with exertional dyspnea since 6 weeks. He had suffered frequent COPD exacerbations in the past but has been free of dyspnea symptoms for several years now. After basic assessment at the outpatient clinic, he was referred for CCTA, which showed a patent LIMA graft on the LAD. A significant lesion was observed in the single SVG on the RCA. SPECT-MPI did not show signs of myocardial ischemia and a normal LV function. After pulmonary assessment, the pulmonologist concluded a stable pulmonary function. However, dyspnea symptoms persisted despite optimal anti-ischemic therapy. Because myocardial ischemia was clinically expected, The Heart Team decided to refer the patient for ICA despite inconclusive stress testing results. Coronary angiography images confirmed a significant graft lesion and the patient was scheduled to undergo PCI of the native RCA. Case 2. A patient with late recurrent angina who underwent CABG 9 years previously, was referred for CCTA after a thorough evaluation of medical history, graft anatomy from the index surgery report, and basic testing. CCTA showed patent grafts (LIMA-LAD, Ao-OM1, Ao-RCA). The native coronary arteries were heavily calcified and obstructive three vessel disease was observed. Myocardial perfusion imaging with CMR showed moderate ischemia in the anterior wall without myocardial fibrosis. The Heart Team subsequently referred the patient for ICA and a significant lesion distal to the anastomosis of the LIMA with the LAD was found. The patient was rescheduled for discussion in the Heart Team and eventually underwent PCI of the distal LAD lesion. ^*^ACS guidelines [116, 117]. (Parts of the figure were drawn by using pictures from Servier Medical Art. Servier Medical Art by Servier is licensed under a Creative Commons Attribution 3.0 Unported License [https://creativecommons.org/licenses/by/3.0/]). Ao ascending aorta, ACS acute coronary syndrome, CMR cardiac magnetic resonance imaging, COPD chronic obstructive pulmonary disease, LAD left anterior descending coronary artery, LIMA left internal mammary artery, LV left ventricle, PCI percutaneous coronary intervention; other abbreviations as in Figs. 1, 2, 3, and 4

Ideally, the Heart Team is provided with information on the timing of recurrent symptoms after initial CABG, cardiac history (including details on left ventricular function and valve abnormalities), CCTA images to establish protected and unprotected coronary territories, and stress testing results to evaluate myocardial ischemia and scar/viability if indicated (Fig. 5). Invasive coronary angiography is the next step in the diagnostic work-up. The recent American clinical practice guideline for the evaluation of chest pain recommend to refer prior CABG patients for ICA to guide therapeutic decision-making when moderate-to-severe ischemia is observed on non-invasive stress testing, or in case myocardial ischemia is clinically expected despite inconclusive stress testing results [15•]. During ICA, the use of contemporary invasive diagnostic tools such as pressure wires and intravascular imaging may enhance physiological understanding of recurrent symptoms after CABG. Indeed, both European and American guidelines recommend ICA in conjunction with invasive functional assessment using fractional flow reserve (FFR) or instantaneous wave-free ratio measurements in patients with and without a prior history of CAD [14•, 15•]. However, data on the value of pressure ratio measurements in patients with grafted coronary arteries, let alone its utility to assess bypass graft lesions, is scarce. In theory, functional assessment of a lesion in a bypass conduit and a native coronary artery relies on similar physiological characteristics, because the ratio of the mean distal pressure to the aortic pressure is used to calculate the FFR and both vessels supply the same myocardial bed [95]. However, competitive flow from the bypass conduit and grafted non-occluded native coronary artery, the presence of coronary collaterals supplying the myocardium subtended by the diseased graft, and differences in viscous friction and flow separation across the graft lesion as a result of different lesion characteristics (friable, diffuse, concentric, and mostly non-calcified) may impact flow hemodynamics and complicate the interpretation of the relationship between flow and pressure in the bypass conduit and grafted native coronary arteries [95–97]. Nevertheless, the possibility to defer from revascularization therapy for hemodynamically non-significant graft lesions is appealing, since revascularization in graft lesions is prone to cause periprocedural complications such as graft dissection, perforation, and distal embolization resulting in slow or no reflow and subsequent periprocedural MI [36]. To date, only few studies investigated the potential application of invasive physiological assessment in bypass graft lesions. Di Serafino et al. compared an angiographic (*N* = 158) with an FFR-based (*N* = 65) PCI approach in patients with an intermediate venous or arterial graft lesion and found that the use of FFR (cutoff ≤ 0.8) to guide revascularization decision-making resulted in better long-term clinical outcomes compared to the use of angiography alone [98]. Almomani and colleagues compared deferral of revascularization for lesions in SVGs and native coronary arteries with an FFR > 0.8 and reported worse prognosis when PCI was deferred for non-significant vein graft disease, which may be related to rapid atherosclerotic graft disease progression and/or different SVG plaque characteristics [96]. Notably, more than half of the included vein graft patients in this study presented with ACS due to graft thrombosis and the results should be interpreted with caution when evaluating post-CABG patients presenting with stable angina. Currently, guideline recommendations on the use of FFR in bypass grafts are lacking and its additive value remains to be established. Coronary flow velocity reserve, an alternative measurement for the functional assessment of coronary lesions using Doppler velocity, has been previously described in vein grafts by Salm et al., who found that the hemodynamic significance of SVG lesions better corresponded with SPECT compared to angiographically derived diameter stenosis percentages [99]. Optical coherence tomography and intravascular ultrasound during ICA may prove useful in the evaluation of atherosclerotic characteristics and plaque features in bypass grafts to enhance the understanding and prediction of graft disease, in conjunction with the estimation of (pharmacological) treatment effects [100, 101]. Future directions in invasive diagnostic management in post-CABG patients may further include the application of FFR equivalents such as quantitative flow ratio, virtual FFR, and FFRangio, which are calculated from invasive coronary angiography images using computational fluid dynamics. These tools are currently being validated in clinical practice to assess hemodynamically significant CAD in native non-grafted coronary arteries, and their value may subsequently be expanded to evaluate intermediate lesions in bypass conduits.

### Management Strategies–Medical Therapy vs. Revascularization

Stable angina patients with prior CABG who are diagnosed with significant graft disease and/or progression of native CAD should in general be referred to the Heart Team for further decision-making on treatment strategy due to the complex nature of repeat revascularization procedures [1•, 15•, 102]. To date, randomized trials comparing optimal medical therapy with repeat revascularization in patients with previous CABG have not been conducted. The Heart Team determines the clinical indication for repeat revascularization based on cardiac symptoms, the area and extent of myocardial ischemia, viability/scar assessment, and coronary angiography. According to the European guidelines on myocardial revascularization, repeat revascularization is recommended (Class I, Level of Evidence B) in patients with severe persistent symptom burden despite optimal medical therapy or a large ischemic myocardial territory (> 10% of the left ventricle) [1•]. Importantly, consistent medication adherence in the post-CABG population was reported significantly lower compared to patients with a history of PCI [103, 104]. Although the exact reason for the reduced medical therapy compliance remains undefined, it may partly explain the observed accelerated atherosclerotic disease burden, the high rate of recurrent angina symptoms, and the frequent need for repeat revascularization therapy in patients who have previously undergone bypass surgery. The first important step in post-CABG patient management is emphasis on secondary prevention and patient counseling to ensure adequate guideline-directed medical therapy and improve medication compliance [105].

### Revascularization Strategies—Redo CABG vs. PCI

In patients in whom the indication to undergo repeat revascularization is established, the Heart Team may triage the patient for redo CABG or PCI. Repeat revascularization procedures in patients with prior CABG are technically more complicated compared to patients without a history of CABG. In case of redo CABG, this is related to post-surgical tissue adhesions in the mediastinum, limited options to select additional grafts after initial bypass surgery, and difficulties to preserve patent conduits during the repeat revascularization procedure. PCI in post-CABG patients is complicated by extensive CAD with complex coronary lesion morphology (calcification, CTOs, and diseased bypass grafts with friable atheromatous plaques) [106]. Indeed, both revascularization strategies are associated with increased risk for adverse events compared to myocardial revascularization in CABG-naïve patients, which may also in part be related to advanced patient age and comorbidities [107–109]. A few studies compared long-term outcomes in patients with prior CABG scheduled to undergo repeat bypass surgery or PCI. The AWESOME (Angina With Extremely Serious Operative Mortality Evaluation) randomized trial and registry experience compared mortality rates between the two treatment arms and found similar survival during 3-year follow-up [4]. In a study by Harskamp et al., the composite endpoint of death, myocardial infarction (MI), and repeat revascularization was similar between redo CABG and PCI in patients presenting with bypass graft failure (51% vs. 57.6%, respectively) at 5-years follow-up [109]. They reported a higher number of periprocedural MI in the redo CABG group (20.5% vs. 8.2%), whereas repeat revascularization rates were higher following PCI (30% vs. 8%) after 5 years. In a more recent observational trial, Mohamed et al. described repeat coronary revascularization strategies and patient outcomes in over 550,000 patients with prior CABG [110]. This study showed that PCI was the selected revascularization strategy in > 90% of cases, although the number of redo CABG procedures did increase between 2004 and 2015. The authors reported more in-hospital major adverse cardiovascular and cerebrovascular events (OR 5.36 95% CI 5.11–5.61), mortality (OR 2.84 95% CI 2.60 − 3.11), acute stroke (OR 2.15 95% CI 1.92–2.41), and all-cause bleeding events (OR 5.97 95% CI 5.44–6.55) in the redo CABG group compared to the PCI group. Indeed, mainly due to the higher risk of periprocedural mortality in patients undergoing redo CABG, contemporary guidelines advocate PCI over redo CABG for repeat revascularization in patients with suitable coronary anatomy (Class II, Level of Evidence C) [1•, 2•]. In patients with diffuse CAD, reduced left ventricular ejection fraction, and extensive disease or occlusion of multiple bypass grafts, in particular in case an IMA graft on the LAD was not previously used or is no longer patent, redo CABG may be considered [2•].

### Revascularization Strategies—Target Vessel Selection for Percutaneous Intervention

Following the decision to refer a patient for percutaneous revascularization, the Heart Team subsequently determines whether PCI of the native coronary artery or the failing bypass graft should be performed. Several observational studies have been conducted to compare bypass graft PCI with native coronary artery PCI and showed an association between graft PCI and increased major adverse cardiac events (MACE) [9••, 111, 112•]. As described previously, graft PCI has been associated with complications such as distal embolization and subsequent no-reflow, PCI-related MI, and rapid target vessel failure. Based on this observational work, the European coronary revascularization guideline advocates PCI of the bypassed native coronary artery over bypass graft PCI, if technically feasible (Class 2A, Level of Evidence C) [1•]. Importantly, PCI of the bypassed native vessel in post-CABG patients may also be challenging, since lesions are often heavily calcified and the prevalence of one or more CTOs in post-CABG patients is reported in over 50% [73]. Notably, results from the PROGRESS-CTO (Prospective Global Registry for the Study of Chronic Total Occlusion Intervention) and RECHARGE (REgistry of Crossboss and Hybrid procedures in FrAnce, the NetheRlands, BelGium, and UnitEd Kingdom) registries showed that CTO PCI in prior CABG patients is linked to lower procedural success rates, increased in hospital-mortality risks and a higher risk of long-term MACE compared to patients without previous bypass surgery [11•, 113]. Rathod et al., on the other hand, suggested that inferior patient outcome following PCI in post-CABG patients is largely attributed to the higher number of comorbidities and once adjusted for these external factors, long-term prognosis in patients with and without prior CABG is similar [111]. Importantly, the revascularization guidelines do not distinguish between occluded and non-occluded grafted native vessels when comparing bypass graft vs. native vessel PCI [1•]. The ongoing PROCTOR (Percutaneous Coronary Intervention of Native Coronary Artery versus Venous Bypass Graft in Patients with Prior Coronary Artery Bypass Graft Surgery) trial (clinicaltrials.gov identifier: NCT03805048) is the first randomized study comparing clinical and angiographic outcomes of native vessel vs. venous bypass graft PCI aiming to further explore the appropriate revascularization strategy for patients presenting with obstructive graft disease [114].

## Conclusions

Patients with previous CABG often present with late recurrent angina and require a systematic approach, taking into consideration the timing of presentation, cardiac history, comorbidities, native coronary anatomy, graft patency, and the presence and extent of myocardial ischemia and viability. A large knowledge base on present-day CCTA demonstrated its use to be essential in the evaluation of graft anatomy and patency. Invasive physiological measurements are increasingly used for revascularization decision-making, yet the evidence is limited in post-CABG patients and treatment is guided by angiographic assessment of lesion severity. Additional physiological studies are warranted to better understand graft hemodynamics. The complexity of revascularization decisions in the post-CABG patient merits careful discussion by the Heart Team. The recommendation to perform redo-CABG or percutaneous revascularization will remain an individualized risk–benefit evaluation. Finally, further studies are needed to extend the scientific framework to support the selection of bypass graft PCI versus native vessel PCI.

## Abbreviations

CABG: Coronary artery bypass grafting
CAD: Coronary artery disease
CCTA: Coronary computed tomography angiography
CMR: Cardiac magnetic resonance imaging
CTO: Chronic total coronary occlusion
FFR: Fractional flow reserve
ICA: Invasive coronary angiography
IMA: Internal mammary artery
MACE: Major adverse cardiac events
MBF: Myocardial blood flow
MI: Myocardial infarction
MPI: Myocardial perfusion imaging
PCI: Percutaneous coronary intervention
PET: Positron emission tomography
RA: Radial artery
SVG: Saphenous vein graft
SPECT: Single-photon emission computed tomography
